# Evaluation of two intervention models on contraceptive attitudes and behaviors among nulliparous women in Shanghai, China: a clustered randomized controlled trial

**DOI:** 10.1186/s12978-017-0331-4

**Published:** 2017-06-15

**Authors:** Yuan He, Ning Zhang, Jue Wang, Na He, Yan Du, Jing-Xin Ding, Ying Zhang, Xiao-Tian Li, Jian Huang, Ke-Qin Hua

**Affiliations:** 1 Department of Gynecology, Obstetrics and Gynecology Hospital of Fudan University, Shanghai, 200011 China; 2 Department of Epidemiology and Health Statistics, Fudan University School of Public Health, Shanghai, 200032 China; 3 Department of Clinical Epidemiology, Obstetrics and Gynecology Hospital of Fudan University, Shanghai, 200011 China; 4 Department of Obstetrics, Obstetrics and Gynecology Hospital of Fudan University, Shanghai, 200011 China

**Keywords:** Nulliparous women, Contraceptive attitudes and behaviors, Community intervention, Clustered randomized controlled trial

## Abstract

**Background:**

With increasing acceptance of premarital sex among young Chinese women, the rates of unintended pregnancies and induced abortions are becoming alarmingly high, suggesting the needs of educating women with adequate contraceptive knowledge and providing them with accessible contraceptive services. Previous studies have shown that knowledge and attitudes towards contraception could be modified through intervention strategies. This study aimed to evaluate the effects of two community intervention models on modifying contraceptive attitudes and behaviors among nulliparous women.

**Methods:**

In this clustered randomized controlled trial, nulliparous women aged 18–40 years from 18 communities were enrolled and randomized to either the traditional community intervention model (TC model) or the more comprehensive new community-based intervention model (NC model) with a ratio of 1:2. Contraceptive attitudes and behaviors were assessed before and after the interventions.

**Results:**

A total of 901 nulliparous women were followed. The most common contraceptive method in both groups was condom (approximately 80%) before or after interventions. The rates of using effective contraceptive methods were very low (<5%) even after the intervention. Comparing the NC with TC group, the adjusted ORs and 95% CIs regarding natural family planning, emergency contraceptive usage and short-acting OCPs were1.53 (95% CI: 1.11–2.13), 2.87 (95% CI: 2.05–4.02), and 2.71 (95% CI: 1.65–4.47), respectively; while the ORs and 95%CIs of gynecological examination and contraceptive use were 2.31 (95% CI: 1.63–3.27) and 2.89 (95% CI: 1.98–4.23), respectively. No statistical significant difference was found for the use of effective contraceptive methods at post-intervention among the two models.

**Conclusions:**

High proportions of nulliparous women held negative attitudes and behaviors towards effective contraceptive methods. The NC model, integrating existing health resources, had more positive influence than the TC model on the favorable contraceptive attitudes and behaviors towards the use of any contraceptive methods, but had limited impact on the use of effective contraceptive methods. Our study suggested the feasibility of applying the NC model in Shanghai. Interventions on contraceptive attitudes and behaviors should base on the existing health service system, synthesize resources and selectively apply to populations with distinct characteristics.

## Plain English summary

With more acceptance of premarital sex among young Chinese women, the rates of unintended pregnancies and induced abortions are becoming alarmingly high, suggesting the needs of educating women with adequate contraceptive knowledge and providing them with accessible contraceptive services. This study aimed to evaluate the effects of two community intervention models on modifying contraceptive attitudes and behaviors among nulliparous women, a particularly vulnerable group.

In this clustered randomized controlled trial, nulliparous women aged 18–40 years from 18 communities were enrolled and randomized to either the traditional community intervention model (TC model) or the new community-based intervention model (NC model) with a ratio of 1:2. Contraceptive attitudes and behaviors were assessed before and after the interventions.

A total of 901 nulliparous women were finally followed. The rates of using effective contraceptive methods were very low (<5%) even after the intervention. Comparing the NC with TC group, the adjusted odds ratios (ORs) and their corresponding 95% confidence intervals (95%CIs) regarding natural family planning, emergency contraceptive usage and short-acting OCPs were1.53 (95% CI: 1.11–2.13), 2.87 (95% CI: 2.05–4.02), and 2.71 (95% CI: 1.65–4.47), respectively; while the ORs and 95%CIs of gynecological examination and contraceptive use were 2.31 (95% CI: 1.63–3.27) and 2.89 (95% CI: 1.98–4.23), respectively.

In conclusions, high proportions of nulliparous women held negative attitudes and behaviors towards effective contraceptive methods. The NC model had more positive effects than the TC model on contraceptive attitudes and behaviors towards the use of any contraceptive methods, but had limited impact on the use of effective contraceptive methods. Interventions on contraceptive attitudes and behaviors should base on the existing health service system, synthesize resources and selectively apply to populations with distinct characteristics.

## Background

Community intervention is a core principle of public health practice, providing community-based contraceptive information and services have been used to access hard-to-reach subjects which started in the 1960s in Indonesia, Korea and have spread into Asia, Latin America and Arica in 1980s [1, 2]. Over 80% of unintended pregnancies in developing countries occur among women with unmet needs for modern contraception [3]. An analysis of national surveys in developing countries showed that 26% (222 million) women had unmet needs for modern methods to avoid unplanned pregnancy, and the absolute number changed little between 2003 and 2012 [4]. China alone accounts for one fifth of all abortions [5]. Sexual and reproductive health problems such as unplanned pregnancies and induced abortions constitute important public health problems in China [6]. The number of induced abortions was approximately 272 million between 1970 and 2010 [7]. One study among 53,652 married women reported the number of induced abortions was 22 per 100 pregnancies [8]. Another cross-sectional survey including 79,174 women who underwent induced abortion from 30 provinces in China reported that 35% of those women had endured first induced abortion and 65% a second or subsequent abortion [9]. One worksite-based survey reported that the unmet needs of contraception in young women ranged from 36.8 to 51.2% among Chinese female migrant workers [10]. Another study found that 49.7% of women undergoing abortions were nulliparous [11]. In particular, the contraception knowledge of the nulliparous was very limited: A 2010 nationally representative survey of 10,966 unmarried Chinese women aged 15 to 24 years showed that 19% of those women were sexually active, but only 4% of those had adequate knowledge about contraception [12]. One study in Guangzhou showed that only 39.3% of a sample of 1,003 migrant women reported having acquired contraceptive knowledge from family planning workers [6]. It is a strange phenomenon that even though the contraceptive use in China was as high as 90 per 100 married women of reproductive age, the induced abortion rate was also high at 23 per 1000 women of the same age group (married or unmarried) [13]. One explanation is that contraceptive services in the Chinese family planning program are not intended for unmarried women; as a result, nulliparous women are usually poorly exposed to such information and have limited access to contraceptive services [14, 15].

Shanghai, with an urban population of 23.8 million by 2012 and half of those from other cities or rural areas, is facing unprecedented challenges of internal migration [16]. Shanghai experienced fast economic growth and reform in the past 30 years, accompanying the economic growth is the dramatic social transformation. Attitudes towards sex and sexual behavior have changed and premarital sex has become more acceptable among young women [7, 17]. The Shanghai government has expanded free family planning services to promote reproductive health, for example, the maternal health centers in public hospitals increased from 10 in 2004 to 24 in 2006 [18]. Nevertheless, the number of induced abortion remains large and most cases are among nulliparous women. In Shanghai, the induced abortion rate of married women was 37.1‰, while the rate of unmarried women was higher ranging between 53.2‰ and 57.7‰ [19]. Other studies show inadequate knowledge and utilization of contraceptive services [20, 21]. Very few migrant nulliparous women reported using free family planning services offered at the factory clinic (5%) or the Family Planning Institute (3%) [20]. A survey conducted in Shanghai reported that 49.1% of pregnant teenagers had experienced contraception failure [21]. Providing accessible contraceptive service to nulliparous women is and still will be a significant challenge.

Nulliparous women are a large and important target population for healthy sexual activities in China. Barriers such as inaccessible service costs and distance, lack of awareness about contraceptive methods, embarrassment, provider attitudes and concerns about side-effects or opposition from male partners limited their access to essential reproductive health information and services [22, 23]. Inadequate knowledge and incorrect perception of contraception is associated with incorrect or inconsistent contraceptive usage which increases the risk of unintended pregnancy [24]. Unintended pregnancies impose serious health and economic costs on both individuals and society. Those women may be at greater risks of physically abusing themselves, suffering economic hardship, failing to achieve their educational and career goals [25]. Improving access to contraceptive information, services and supplies is an effective way to reduce the demand for abortion and improve the general health of women [3, 26]. It is crucial to find a suitable intervention model to improve attitudes and behaviors of contraception among nulliparous women.

The main objectives of the current study are to: (1) evaluate the effects of two existing intervention models on contraceptive attitudes and behaviors among nulliparous women (2) determine the appropriate contraception intervention model for nulliparous women during the population structure transition in Shanghai.

## Methods

### Trial conduct and participants

**Fig. 1:**
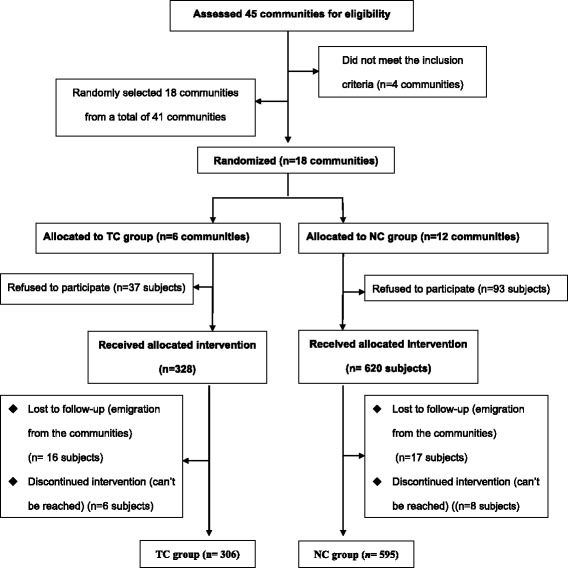
Flow diagram of study population enrollment

This study was part of the community-based reproductive health projects on women during their reproductive period (the projects were divided into pre-conception, prenatal and child-born sub-projects) [27]. This prospective, parallel, randomized controlled study was carried out from 1st June 2013 to 30th December 2013 with a total duration of 6 months, in Shanghai, China. A multistage stratified clustered systematic sampling method was used to allocate the two arms. Seventeen districts in Shanghai were divided into low, medium and high groups according to their economic levels as defined by per capita gross domestic product (GDP) per district in 2012. Two districts from each group were selected randomly (Low: Jiading, Minhang; Medium: Yangpu, Changning; High: Jingan, Huangpu). Eighteen representative communities were selected from the total of 41 communities in these six districts and were randomized into the intervention group (the new community-based intervention model, NC model) and the control group (the traditional community intervention model, TC model) at an intervention-to-control group ratio of 2:1 (Fig. 1). The unit of randomization was community; stratified variables included the size of the female population in 2012, the distance from the maternal and child health hospital in each district.

Sixty households with nulliparous women were selected from each community.

The households were registered by the community family planning workers as a routine work except the households who lived in the community for less than 6 month. The random assignments, using a random number table, were generated by blinded study staff from Epidemiology and Biostatistics department in Obstetrics and Gynecology Hospital of Fudan University. The final households with nulliparous were selected from the existing frame of households, when two or more nulliparous women are in a household, only one was randomly selected in order to avoid intra-class correlation. Finally, eligible nulliparous women from each selected households were asked if they wished to join the study. The recruitment process was conducted by community reproductive health workers, and lasted for about one month. In total, 1078 women were screened, 37 nulliparous women in TC group and 93 subjects in NC group refused to participate in the study, Of the 948 enrolled subjects, 33 subjects removed from the living communities and 14 subjects couldn’t be reached because of personal requirement. Finally, a total of 901 nulliparous women were followed-up (306 subjects in the TC group and 595 subjects in the NC group, Fig. 1).

The study protocol conformed to the ethical guidelines of the 2000 Declaration of Helsinki and was approved by the Institutional Review Board at Obstetrics and Gynecology Hospital of Fudan University. All participants provided written informed consent. The trial was monitored by an independent data and safety monitoring board consisting of 5 external epidemiologists and biostatisticians appointed by Obstetrics and Gynecology Hospital of Fudan University.

### Inclusion criteria

Women meeting the following eligibility criteria were included in the study: (i) living in the selected community (both the local residents and immigrants); (ii) giving informed consent; (iii) born in mainland China; (iv) 18–40 years of age; (v) nulliparous.

### Intervention

The TC model utilizes traditional contraceptive strategies and is consisted of: (i) provision of information through group education; (ii) a few government-funded free training for community doctors (1–2 times training about professional skills); (iii) basic reproductive and contraceptive services supported by the government, which are free for married women and provided to unmarried women at a very low price.

The NC model is a newly developed community-based intervention. In addition to the strategies used in the TC model, the following items are also included in the NC model: (i) Distribution of a 48-page booklet entitled *The Reproductive Health Handbook* to each participant. This booklet includes 5 pages of contraceptive information, including contraceptive knowledge (mainly including condoms, oral contraceptives pills [OCPs], intrauterine device [IUD], implants, contraceptive patch, vaginal contraceptive ring, spermicides, diaphragm, natural family planning), the effectiveness of emergency contraception (what is emergency contraception, where can you get emergency contraception, how to take emergency contraception pills, the efficacy and the side effect of emergency contraception), favourable attitudes and behaviors towards contraceptive use, method of provision, the methods and side effect of induced abortion, resource accessibility; The booklet is designed by a group of 22 gynecologists, epidemiologists, and maternal and child health professionals for family reproductive health. (ii) Providing each subject with counseling services which include basic reproductive health information, the risk of pregnancy if contraception was used incorrectly, the contraceptive benefits on birth control, contraceptive methods, side effects of different contraception and how to handle the problem, contraceptive recommendation and specific plan for at least 10 min by a six-member panel, the six-member panel was consisted of community health workers, community doctors and a doctor from the maternal and child health hospital in the district. (iii) Two-day lecture was conducted by gynecologist from tertiary hospital for the six-member panel, the content of the health lectures focused primarily on the reproductive health knowledge, contraceptive guideline, contraceptive resourse, contraceptive referral system, counseling skills, etc. (iv) Four-hour group education, including contraceptive information, the prevention of sexually transmitted disease and public contraceptive resource, was hold for nulliparous women in each selected district by gynecologists from a tertiary hospital. All experts underwent standardized training provided by the project group.

### Data collection and evaluation

All participants were assessed with a structured questionnaire through face-to-face interviews before and after the 6-month intervention. The interviews were conducted by 30 trained interviewers at the participants’ home or at the community survey sites. All interviewers received an 8-h training session before the intervention. Those who were not able to complete the interview within two weeks were excluded from the study.

The questionnaire was designed by three investigators (KH, NZ, YH) based on published literatures [23, 28]. After two rounds of discussion among the investigators and one round of discussion with external experts (epidemiologists, obstetrician and community doctors), the final version of the questionnaire consists of four sections and a total of 22 items. The first section includes socio-demographic information and lifestyle factors such as smoking and drinking. The second section is baseline reproductive health history which contains abortion history, pregnancy plan in the next year, any previous or current gynecological diseases. The third section collects information about attitudes towards contraception, which include the perception of contraception time after abortion, necessity of contraception, harm of abortion, natural family planning, emergency contraception, use of short-acting OCPs and condoms. The fourth section focuses on contraceptive behavior currently, including use of any types of contraceptive methods such as condoms, natural methods, IUD, OCPs, emergency oral contraceptives, sterilization, injections, implants, spermicidal, etc; use of more effective contraceptive methods such as condoms, IUD, OCPs, implants, injections; gynecological examination.

### Statistical analysis

Based on previous studies of traditional community interventions, it was estimated that 60% of women in the control group and 70% of women in the intervention group would use contraception [28]. At an intervention-to-control-group ratio (NC: TC) of 2:1, allowing a drop-out rate of 10%, a total sample size of 900 (600 in the intervention group and 300 in the control group) was adequate to detect a 10% difference with 80% power at the significance level of 5%.

Categorical variables are presented as frequencies and proportions, and the continuous variables are presented as means and standard deviations. Chi-square test or Fisher’s exact test was used to compare categorical variables. Univariate and multivariate logistic regressions were applied to estimate the odds ratios (ORs) and 95% confidence intervals (95% CIs) of NC and TC groups before and after the interventions, as well as NC versus TC model after the intervention. Adjusted variables in the multivariate logistic regression model were age, education, salary, birthplace, sexual history, and pregnancy plan for the next year. All significance tests were two sided; and *P* value of <0.05 was considered statistically significant. All statistical analyses were performed using STATA 12 software (Stata Corporation, College Station, Texas).

## Results

### Demographic characteristics and baseline reproductive health

A total of 901 nulliparous women aged 18–40 were assessed before and after the intervention. Table 1 presents the demographic characteristics. In brief, among all participants, 68.2% were between 18–29 years old, 87.9% received college or higher level education, 76.6% of the participants were born in Shanghai, and the ratio of married versus unmarried women was 3:2. Of all participants, 13.4% had a history of abortion, and half of the participants had pregnancy plans for the next year. About one third (31.0%) of the women had gynecological disease in the previous six months, while only 20.2% of them had sought help from doctors. There was no significant difference between the two groups for all demographic characteristics and baseline reproductive health information except for educational level and average monthly income, which are both slightly higher in the NC group.

**Table 1:** Demographic characteristics and baseline reproductive health information

Characteristics	TC (*n* = 306)	NC (*n* = 595)	ALL	P*
*Demographic characteristics*				
Age (years)				
Mean ± SD	28.05 ± 4.24	28.03 ± 3.76	28.04 ± 3.93	0.93
Educational level				
High/vocational school or lower	48 (16.0%)	58 (10.0%)	106 (12.1%)	**0.01**
College or higher	253 (84.0%)	518 (90.0%)	771 (87.9%)	
Marital status				
Married/divorced/separated	150 (59.3%)	345 (65.7%)	495 (63.6%)	0.08
Unmarried	103 (40.7%)	180 (34.3%)	283 (36.4%)	
Birth place				
Shanghai	189 (74.7%)	407 (77.5%)	596 (76.6%)	0.38
Other regions	64 (25.3%)	118 (22.5%)	182 (23.4%)	
Average monthly income (RMB)				
< 5000	144 (56.9%)	211 (46.8%)	427 (55.0%)	**0.01**
≥ 5000	109 (43.1%)	240 (53.2%)	349 (45.0%)	
Smoking	35 (22.3%)	74 (20.1%)	109 (20.8%)	0.57
Drinking	5 (4.5%)	9 (3.1%)	14 (3.5%)	0.52
*Reproductive history*				
History of abortion (times)				
0	222 (87.7%)	452 (86.1%)	674 (86.6%)	0.53
1-2	31 (12.3%)	73 (13.9%)	114 (13.4%)	
Pregnancy plan in the next year	113 (44.8%)	252 (51.8%)	365 (49.4%)	0.08
Gynecological diseases in the previous six months	85 (28.2%)	187 (32.4%)	272 (31.0%)	0.21
Seeing a doctor because of gynecology diseases	58 (19.3%)	119 (20.6%)	177 (20.2%)	0.26

*P from comparisons of TC group and NC group, Statistically significant (*P* < 0.05) results are indicated in bold style

### Intervention effects

Our study found that participants from both TC and NC groups had high perceptions of the need for contraception (all > 92%) at baseline and post-intervention. On the contrary, the rates of favourable attitude related to using short-acting OCPs were low in both groups before and after the interventions. The rates of using effective contraceptive methods such as short-acting (2.7% in TC group and 3.1% in NC group) or long-acting OCPs (1.1% in TC group and 0.5% in NC group) were very low even after intervention. In addition, the rates of using natural family planning (around 20%) were alarmingly high in both groups even after the interventions, which warrants particular attention since neither are effective contraceptive methods. The most common contraceptive method was using condom (approximately 80%) both before and after interventions in the two groups. Compared to the TC model, more participants in the NC model considered natural family planning (NC group versus TC group = 56.9% versus 44.8%) and emergency contraception (NC group versus TC group = 28.5% versus 23.2%) were not reliable after the intervention (Table 2).

**Table 2:** Contraceptive attitudes and behaviors before and after program implementation among nulliparous women

Characteristics	TC model		*P* Value	NC model		*P* Value
	Before	After		Before	After	
Attitudes						
Appropriate contraception time after abortion	147 (58.8%)	179 (71.6%)	**0.003**	289 (60.3%)	377 (82.0%)	**<0.001**
Contraception is necessary for women with no attention of getting pregnant	232 (92.8%)	235 (94.0%)	0.59	453 (94.6%)	446 (97.0%)	0.07
Natural family planning is not reliable	106 (42.4%)	112 (44.8%)	0.59	203 (42.4%)	263 (56.9%)	**<0.001**
Emergency contraception is not reliable	44 (17.6%)	58 (23.2%)	0.12	134 (28.0%)	131 (28.5%)	0.85
Emergency contraception pills cannot be taken more than 3 times in a year	76 (30.4%)	82 (32.8%)	0.56	178 (37.2%)	273 (59.4%)	**<0.001**
Accepting short-acting OCPs	54 (17.9%)	55 (18.3%)	0.92	100 (17.3%)	145 (26.0%)	**<0.001**
Using condom alone is not enough to prevent reproductive tract infection	136 (53.3%)	176 (68.2%)	0.94	258 (53.0%)	312 (66.5%)	**<0.001**
Behaviors						
Underwent gynecology examinations in the previous six months	154 (51.2%)	155 (51.5%)	0.93	347 (60.1%)	403 (72.2%)	**<0.001**
Current contraceptive methods ∆						
*Short-acting OCPs*	4 (2.2%)	5 (2.7%)		9 (2.2%)	13 (3.1%)	
*Natural family planning*	34 (18.5%)	39 (21.0%)		79 (19.3%)	82 (19.4%)	
*External ejaculation*	13 (7.1%)	10 (5.4%)		55 (13.4%)	58 (13.7%)	
*Emergency contraception*	7 (3.8%)	10 (5.4%)		5 (1.2%)	5 (1.2%)	
*Condoms*	155 (84.2%)	144 (77.4%)		340 (82.9%)	349 (82.5%)	
*Long-acting OCPs*	2 (1.1%)	2 (1.1%)		3 (0.7%)	2 (0.5%)	
*IUD*	7 (3.8%)	9 (4.8%)		4 (1.0%)	4 (1.0%)	
*Sterilization/implant,*etc.	8 (4.4%)	12 (6.5%)		17 (4.1%)	17 (4.0%)	
Use of any contraceptive methods	184 (61.1%)	186 (61.8%)	0.87	410 (71.1%)	423 (75.8%)	0.07
Use of effective contraceptive methods	164 (54.5%)	156 (51.8%)	0.51	350 (60.7%)	359 (64.3%)	0.20

Statistically significant (*P* < 0.05) results are indicated in bold style. *Analyses did not include missing values

∆Participants could select more than one response, so the percentages sum to more than 100%

The rates of favourable contraceptive attitudes and behaviors on OCPs, emergency contraception, condom and behavior among the NC group all increased (ranging from 0.6 to 22.2%) after the intervention, while the rates increased less in the TC group after the intervention (ranging from -2.7 to 14. 9%). The average rate of effective contraceptive attitudes and behaviors increased 3.3% in the TC group after intervention, while the increase was 9.4% in the NC group, almost three times of the TC group (Table 2). However, different proportion of contraceptive attitudes and behaviors existed at baseline, 28% of subjects in NC group thought that emergency was not reliable but the proportion was only 17.6% in TC group, the rate of using any contraception methods also had a gap in the two groups (71.1% in the TC group and 61.1% in the NC group). Complex factor affect the attitudes and behaviors, unbalanced rate of individuals on education and income may due to the difference of the contraceptive attitudes and behaviors in baseline.

Univariate and multivariate logistic regressions were used to estimate the effectiveness of the interventions on contraceptive attitudes and behaviors (Table 3). For the TC model, there were no statistically significant adjusted ORs. For the NC model, the adjusted ORs were significant for the following items: natural family planning is not reliable, appropriate contraception time after abortion, emergency contraception pills cannot be taken more than 3 times in a year, accepting short-acting OCPs, using condom alone is not enough to prevent reproductive tract infection, underwent gynecology examinations in the previous six months, and use of any contraceptive methods. For NC model versus TC model after the interventions, the statistically significant adjusted ORs for natural family planning is not reliable (adjusted OR = 1.53, 95%CI: 1.11–2.13), emergency contraceptive usage (adjusted OR = 2.87, 95%CI: 2.05–4.02), accepting short-acting OCPs (adjusted OR = 2.71, 95%CI: 1.65–4.47) and having gynecological examinations (adjusted OR = 2.31, 95%CI: 1.63–3.27). No significant differences were found at baseline with regard to use of any contraceptive methods and use of effective contraceptive methods between the NC model and TC model after controlling the background characteristics. However, the participants in the NC model had more than twice the odds of use of any contraceptive methods than those in the TC model at post-intervention (adjusted OR = 2.89, 95%CI: 1.98–4.23). The NC model had significant influence on the favourable awareness and attitude of contraceptive methods, as well as the use of any contraceptive methods. However, there was no statistical significance difference among the two models in the use of effective contraceptive methods at post-intervention (adjusted OR = 1.18, 95%CI: 0.69–2.04).

**Table 3:** Odds ratios and 95% confidence intervals of the contraceptive attitudes and behaviors between before and after intervention and comparison between intervention packages

Characteristics	TC model		NC model		NC vs. TC	
	Crude OR (95%CI)	Adjusted OR (95%CI)	Crude OR (95%CI)	Adjusted OR (95%CI)	Crude OR (95%CI)	Adjusted OR (95%CI)
Attitudes						
Appropriate contraception time after abortion	1.77 (1.21–2.56) **	1.87 (1.26–2.78)*	2.99 (2.21–4.03) ***	2.98 (2.19–4.05) ***	1.80 (1.25–2.59) ***	1.51 (1.02–2.22)
Contraception is necessary for women with no attention of getting pregnant	1.22 (0.60–2.47)	1.27 (0.64–2.63)	1.83 (0.94–3.59)	1.59 (0.80–3.15)	2.03 (0.97–4.28)	1.73 (0.77–4.90)
Natural family planning is not reliable	1.10 (0.77–1.57)	1.12 (0.79–1.61)	1.80 (1.38–2.33)***	1.83 (1.40–2.38)***	1.63 (1.19–2.22)**	1.53 (1.11–2.13)*
Emergency contraception pills cannot be taken more than 3 times in a year	1.12 (0.77–1.63)	1.15 (0.78–1.70)	2.49 (1.90–3.21)***	2.53 (1.94–3.31)***	3.00 (2.17–4.13)***	2.87 (2.05–4.02) ***
Accept short-acting OCPs	1.02 (0.68–1.55)	1.04 (0.60–1.92)	1.67 (1.26–2.23)***	2.71 (1.85–3.97)***	1.57 (1.11–2.22) *	2.71 (1.65–4.47) ***
Using condom alone is not enough to prevent reproductive tract infection	1.88 (1.31–2.69) ***	1.86 (1.28–2.68)*	1.76 (1.36–2.29)***	1.85 (1.41–2.43) ***	0.93 (0.67–1.28)	1.01 (0.71–1.44)
Behavior						
Underwent gynecology examinations in the previous six months	1.01 (0.74–1.40)	1.07 (0.73–1.57)	1.72 (1.34–2.21)***	1.92 (1.44–2.55) ***	2.45 (1.83–3.28) ***	2.31 (1.63–3.27)***
Use of any contraceptive methods	1.03 (0.71–1.83)	0.93 (0.60–1.43)	1.28 (0.71–1.64)	1.41 (1.01–1.96) *	1.94 (1.39–2.33)***	2.89 (1.98–4.23)***
Use of Effective contraceptive methods	0.9 (0.65–1.24)	0.56 (0.27–1.14)	1.17 (0.65–1.24)	0.89 (0.58–1.35)	1.68 (1.27–2.25)***	1.18 (0.69–2.04)

Variables were adjusted by age, education, average monthly income, birthplace, sexual history, pregnancy plan in the next year; *P < 0.05, **P < 0.01, ***P < 0.001

Analyses did not include missing values

*OCPs* oral contraceptive pills, *OR* odds ratios, *95% CI* 95% confidence interval

## Discussion

Many young people in developing countries are increasingly exposed to risks of unprotected sexual activities, which impose serious public health problems to the society [29]. In this study, the abortion rate for nulliparous women was nearly 14%, while for those with gynecological diseases only a small proportion (20%) had sought help from a doctor in the previous six months. This study also showed that a considerable proportion of women chose less-effective contraceptive methods such as natural family planning and external ejaculation even after the intervention. On the contrary, the rate of using effective contraceptive methods such as IUD, shot- and long-acting OCPs is very low (lower than 5%) even after the intervention. The relatively high proportions of women who held negative attitudes towards effective contraceptive methods and had low medical service utilization highlight the importance of education and consultation about appropriate contraception.

There are several community intervention studies conducted in both developed (United States, Australia) and developing countries (China, Ghana) to promote favourable contraceptive use. Those studies have targeted different populations, including migrant women, adolescents, adults, post-abortion women, and mixed populations (both adults and adolescents) with a relatively wide age range (13–48 years old), through a variety of media and interactive methods [21, 23, 24, 27–32]. Those intervention programs can be classified into four types: facility based, out-of facility based, interventions to reach marginalized or vulnerable populations, and interventions to generate demand and/or community acceptance [33]. The shift in the past decade from the most effective method towards barrier methods might have led to increases of unintended pregnancies in women using modern methods [4]. Whether married or not, nulliparous women have particular needs in contraception because they are more likely to have unprotected and non-consensual sex, and usually lack the information and services needed to protect themselves [29]. However, researches of community education for contraceptive use among nulliparous women are limited and highly variable [15, 16, 24, 34]. Different studies have shown improvement of contraceptive knowledge and attitude among the intervention groups [18, 35]. Our study, in particular, demonstrated that a new contraceptive intervention model, the NC model, had more positive influences on favourable contraceptive attitudes and behaviors toward the use of any contraceptive methods among nulliparous women than the TC model, even though no significant change was observed in using effect contraceptive methods. The base theories of the NC model were the Health Belief Model and Social Cognitive Theory. Current behaviors, thoughts, emotions and environment all interact to affect new behavior, individuals will take action to prevent abortion if they believe they are susceptible or the benefits of action outweigh the costs [36]. Though the rate of using specific contraceptive methods increased slightly in the NC models in pre- and post-intervention period, the difference was statistically significant after adjustment for baseline characteristics. The possible mechanisms of the NC model could attribute five points:(1) The NC model, synthesizing community health resources and district family planning system to provide counseling and medical services, enhance the accessibility and quality of family planning services for nulliparous women in the community. Research showed that contraceptive counseling and services led to more contraceptive use than no provision [37]. (2) The NC model generates a more diversified access to contraception-related information than TC model for nulliparous women. Evidence from previous studies suggests that women who have access to get contraceptive information were more likely to use contraception than others [38]. (3) The training for community worker and the cooperating group from tertiary, district and community enhance the accountability and responsibility of service providers in community.(4) The NC model reduce inequalities in public health more generally, either married or unmarried nulliparous women acquired the same intervention, the intervention may enhance community awareness, understanding, and acceptance of contraception. (5) The NC model, providing a close and sustainable communication with the subjects, may create a more stable and trusted relationship between the community residents and professional health-service. However, there were no significant changes on the effective contraceptive behavior in the NC group, possibly because 6 months may not be long enough to initiate and sustain a change on the effective contraceptive method, and the intervention alone may not be sufficient to increase the use of effective contraceptive methods. In addition, socioeconomic characteristics especially educational level and income should also be taken into consideration.

Women’s contraception needs change during the courses of their lives, and differ by developmental stage, education, socioeconomic status, marital status and other individual features [39, 40]. Nulliparous woman’s understanding and attitude of information related to contraception may play a critical role, either directly or indirectly, on contraception use and the fertility intention. We may infer from the existing result that the nulliparous women in the NC model tend to have more awareness and autonomy in choosing contraceptive methods to reduce abortion risks and determine the pregnancy plan. Though a part of subjects who had the pregnancy plan in the next year in the study, the intervention also may have a influence on their prevention of the sexually transmitted disease, the contraceptive attitude and behavior in the child-born stage. However, further influence on fertility preference among the nulliparous women couldn’t be obtained due to the lack of follow-up in the study.

Costs and time on contraception, fear of side effects, misconceptions, lack of support from partner were barriers existing in the NC model, premarital sex was considered a moral issue which was taboo in Chinese traditional culture, stigma in reputation was also a hindrance to get contraception for unmarried nulliparous women. Many barriers such as limited access to medical care or the cost of health services are unlikely to amend in a short period of time. Therefore, easily implemented and accessible intervention strategies such as the booklet campaign proposed by this study could be recommended to nulliparous women. In addition, the existing reproductive health system should focus more on nulliparous women who are migrants, unmarried, and with less education and lower income. Studies have demonstrated that using structured counseling, phone calls, and other new technologies improved compliance with contraceptive use [41, 42]. About 73.1% of Shanghai residents used internet by the end of 2015 [43]. It is appealing to conduct contraceptive interventions through new media such as Weichat, Micro Blog, and Instant Messaging. Furthermore, it is essential to provide accessible medical services and improve the referral system to promote contraceptive use.

Our studies have several limitations. First, our study was conducted in Shanghai, a city with well-developed economic and health infrastructure, therefore our findings may not be generalized to rural areas in China. Second, information collected through self-reporting may introduce certain biases. For example, the low response rate in OCPs use may due to the lack of knowledge regarding certain contraceptive items. Third, the research was based on the assumption that the unmarried women were sexually active and potentially in need of contraception, this assumption may overestimate the effect of the intervention. Last but not least, the NC model is a rather dense program which requires intensive training, sustainable financial support, and long-time commitment from all involved parties. How to best carry out such a program is challenging and warrants future studies.

## Conclusions

To conclude, the NC model increased the rates of favourable contraceptive attitudes and behaviors towards the use of any contraceptive method, but it had limited impact on the use of effective contraceptive methods. Although further confirmation are needed from future prospective studies, the NC model maybe a suitable intervention strategy to improve the contraceptive attitudes and behaviors among nulliparous women. Future research should further investigate how to effectively synthesize the service and family planning system.

## Abbreviations

95% CIs: 95% confidence intervals
GDP: Capita gross domestic product
IUD: Intrauterine device
NC model: The new community-based intervention
OCPs: Oral contraceptive pills
ORs: Odds ratios
TC model: The traditional community intervention model
